# Stress and psychological factors before a migraine attack: A time-based analysis

**DOI:** 10.1186/1751-0759-2-14

**Published:** 2008-09-18

**Authors:** Masahiro Hashizume, Ui Yamada, Asako Sato, Karin Hayashi, Yuichi Amano, Mariko Makino, Kazuhiro Yoshiuchi, Koji Tsuboi

**Affiliations:** 1 Department of Psychosomatic Medicine, Faculty of Medicine, Toho University, 6-11-1 Omorinishi, Ota-ku, Tokyo, 143-8541, Japan; 2 Department of Stress Sciences and Psychosomatic Medicine, Graduate School of Medicine, the University of Tokyo, 7-3-1 Hongo, Bunkyo-ku, Tokyo 113-8655, Japan

## Abstract

**Background:**

The objective of this study is to examine the stress and mood changes of Japanese subjects over the 1–3 days before a migraine headache.

**Methods:**

The study participants were 16 patients with migraines who consented to participate in this study. Each subject kept a headache diary four times a day for two weeks. They evaluated the number of stressful events, daily hassles, domestic and non-domestic stress, anxiety, depressive tendency and irritability by visual analog scales. The days were classified into migraine days, pre-migraine days, buffer days and control days based on the intensity of the headaches and accompanying symptoms, and a comparative study was conducted for each factor on the migraine days, pre-migraine days and control days.

**Results:**

The stressful event value of pre-migraine days showed no significant difference compared to other days. The daily hassle value of pre-migraine days was the highest and was significantly higher than that of buffer days. In non-domestic stress, values on migraine days were significantly higher than on other days, and there was no significant difference between pre-migraine days and buffer days or between pre-migraine days and control days. There was no significant difference in the values of domestic stress between the categories. In non-domestic stress, values on migraine days were significantly higher than other days, and there was no significant difference between pre-migraine days and buffer days or between pre-migraine days and control days.

There was little difference in sleep quality on migraine and pre-migraine days, but other psychological factors were higher on migraine days than on pre-migraine days.

**Conclusion:**

Psychosocial stress preceding the onset of migraines by several days was suggested to play an important role in the occurrence of migraines. However, stress 2–3 days before a migraine attack was not so high as it has been reported to be in the United States and Europe. There was no significant difference in the values of psychological factors between pre-migraine days and other days.

## Introduction

Drug therapy for migraines has progressed rapidly in recent years. Especially, improvement in the treatment of acute phase migraine has been remarkable with the launch of triptans. No treatment that can completely cure a migraine attack has been established. However, preventive treatments have been applied to migraine with frequent attacks.

It is important for drug-free, preventive treatments of migraine to remove or avoid the existing causes of migraine. Thus, many studies have been done of the precipitating factors[1-9], including stress, menstruation, changes in weather, alcohol consumption, specific foods, sleep disorder, light, noise, odor, bathing, and drugs[1,4,5].

Recent knowledge about the relationship between migraine and stress suggests that stress is a key precipitating and aggravating factor that causes or worsens migraine [2]. However, it is questionable if such relationship is specific to migraine. This is because it was reported that stress caused migraine and tension-type headache [2] and that the severity of stress in daily life was lower in migraine patients than in tension-type headache or combined-type headache patients [10].

It is suggested from clinical experience that there is a time lag of a few days between stress and migraine attack. Recent prospective studies, which assess the causal relationship between migraine and time, have shown that migraine occurs not during changes in stress but from a few hours to one or two days after stress [11-15].

There are reports that mood changes such as alertness, tension, depressive tendency, irritability and fatigue, as well as stress, also went a few days ahead of migraine attack [11,13].

The morbidity of migraine tends to be lower in Asia than in the United States and Europe. This is assumed to be related to differences in lifestyle and race [16]. There are very few reports of the relationship between migraine and stress in Asian countries.

The objective of this study is to chronologically examine the relationship between migraine and stress and mood changes in a Japanese population.

The relationship of migraine to stress has been treated from different points of view in the past. In most prior studies, however, stress was treated comprehensively with no categorization. If categorized, stress was divided into only two groups: stressful events and daily hassles, and stress details remain indefinite. A few studies showed the association between the stress at work and migraine [17,18]. However, the association between the stress at home and migraine was not discussed. Under such a background, we examined "domestic" and "non-domestic" stresses.

Both "domestic" and "non-domestic" stress were categorized into stressful events and daily hassles, as in conventional studies, and into domestic stress and non-domestic stress. In addition to depressive tendency and irritability, we also examined anxiety and sleep conditions that have a close relationship to migraine.

## Methods

### Subjects

Nineteen outpatients (17 females and 2 males) in the Department of Psychosomatic Medicine of Toho University were registered. The study was conducted from April 2003 to August 2007. Subjects were volunteers who intended to participate in the study after understanding the objectives and details of the study: Written informed consent was obtained from all participants. The eligibility age was from 15 to 60 years. If subjects were minors, the objectives and details of the study were also explained to their guardians, and written informed consent was also obtained from them. For the diagnosis of migraine, the International Classification of Headache Disorders:2nd edition (ICHD-II) was used [19].

No subject had a physical disorder other than migraine. Subjects with headache and drug abuse-associated headache were excluded. The data of three subjects was censored (one; due to temporal inconveniences and two due to inappropriate data), leaving the data of 16(14 females and 2 males) subjects available for analysis. The mean age was 32.0 years and the range was 19 to 48 years. Background information is shown in Table 1.

**Table 1:** Demographic and characteristics of migraine subjects

	migraine patients (N = 16)
Age (years)	
average ± SD, range	32.0 ± 9.0, 19–48
Males/females	2/14
Occupation	
office worker	8/16
part-timer	5/16
housewife/part-timer	3/16
Duration of disease (months)	
average ± SD, range	115.4 ± 78.1, 12–264
Frequency of attacks (number of attacks per month)	
average ± SD, range	5.3 ± 2.7, 2–10
Pain characteristics	
With aura	8/16
Pulsating	15/16
Photophobia	14/16
Phonophobia	14/16
Family history (+)	4/16
Headache VAS score	
average ± SD, range	7.8 ± 10.7, 0–40
Having a mood disorder	4/16 (major depression 4)
Having a anxiety disorder	2/16 (panic disorder 2)

### Headache diary

The subjects were required to fill out a headache diary four times a day (on awakening, 13:00, 18:00 and at bedtime) for two consecutive weeks. (In conventional studies of migraine, subjects were usually required to fill out a headache diary for one month or more. However, focusing on each event of migraine attack rather than each subject in this study, we requested them to fill out a headache diary for two weeks to reduce their burden.)

The 40 mm visual analogue scale (VAS) was used to assess the severity of headache, stressful events, daily hassles, domestic and non-domestic stress, sleep quality (sleep quality recorded only in recordings on awakening), sleepiness, anxiety, depressive tendency, and irritability. Stressful events are usually assessed by their number; however in this study, to distinguish them from daily hassles, we assumed that the subjects felt the events to be stress when they wrote them in the diary. As a result, daily hassles were usual stress events which occurred on the day when the subjects wrote, and the stressful events were stress in the past and future.

A higher VAS score on an item indicates a strong tendency to meet each item except sleep quality. For sleep quality, a lower VAS score indicates a more severe disturbance of sleep. Sleep quality was recorded on awakening.

The subjects were also asked about menstruation, features of headache and other symptoms. We asked the patients in the daily diary to indicate the following features and associated symptoms of the headache: pulsating quality of the pain, nausea and/or vomiting and sensitivity to light and/or noise. The subjects were required to answer "Yes" or "No".

We categorized the contents of the headache diaries according to the severity of headache and other symptoms as below.

(1) Migraine days: days when the subject had a headache that meets all of the three criteria below

1) Severity of headache: 24 mm or higher on VAS

2) Headache causing pulsating or difficulties in daily living

3) Headache causing nausea or hypersensitivity to light and sound

(2) Pre-migraine days: 1–3 days before migraine, excluding migraine days and their following days

(3) Buffer days: excluding migraine days and pre-migraine days, days of 5–24 mm headache severity, menstruation days, and days following the days of migraine till two days before the menstruation (if the migraine days are consecutive, days following the last day)

(4) Control days: days other than (1) to (3) above

The categorization method of headaches and the range 5 mm to 24 mm were **as in **prior studies [13,14].

### Statistical analyses

The data used in the present study have a structure in which recordings are nested within patients, and the numbers of recordings for each category were different among patients. Thus, we investigated the difference in the subjective symptoms recorded by the diary among the four categories of diary days modeling with the Tukey-Kramer's multiple comparison test (SAS PROC MIXED procedure [20]. In addition, the effect of menstruation was controlled because menstruation might affect subjective symptoms. Thus, the model used in the present study was expressed as follows:

Level 1 equation:

*Y*_*ij *_= *π*_0*i *_+ *π*_1*i *_*Days*_*ij *_+ *π*_2*i *_*Menstruation*_*ij *_+ *ε*_*ij*_

Level 2 equations:

*π*_0*i *_= *γ*_00 _+ *ζ*_0*i*_

*π*_1*i *_= *γ*_10_

*π*_2*i *_= *γ*_20_

where Y_*ij *_is each subjective symptom for the *i*th patient; *Days*_*ij *_corresponds to one of the four categories of days, and Menstruation_*ij *_is the corresponding dichotomous variable, "menstruation day" or not. *π*_0*i *_is the individual *i*'s true value of mean each subjective symptom when all predictors are zero (intercept). *γ*_00 _is the average true value of the mean of each subjective symptom when all predictors are zero. *π*_1*i *_is the effect of each days category on each subjective symptom of the individual *i*, *γ*_10 _is the average effect of each days category. *ε*_*ij *_and *ζ*_0*i *_are residuals at each level. Including *ζ*_0*i *_in the equation means that the intercept is modeled as random, which suggests that the intercept could vary across patients. Equations without residuals means that the effect of each predictor is modeled as fixed.

## Results

### 1) Incidence of migraine

The aggregate of days of measurement were 221 days in total: 43 migraine days (19.5%), 32 pre-migraine days (14.4%), 80 buffer days (36.2%) and 66 control days (29.9%). There were 27 migraine attacks in total. Two of the subjects had no migraine attack. The highest number was five migraine attacks for one subject. The mean number of migraine attacks was 1.6 ± 1.4.

### 2) Relationship to stress

Table 2 shows the totaled results of stressful events and daily hassles by categorized days, based on the diaries.

**Table 2:** Mean scores for stress types for each of the days

	Migraine days N = 172 Mean (S.E.) effect	Pre-migraine days N = 128 Mean (S.E.) effect	Buffer days N = 320 Mean (S.E.) effect	Control days N = 265 Mean (S.E.) effect
Headache severity	17.1 ± 1.4^**1 **2 **3^	7.0 ± 1.4^**1^	7.2 ± 1.3^**1^	4.2 ± 1.4
Stressful events	12.3 ± 1.5^*2^	11.7 ± 1.5	9.7 ± 1.4^**1^	13.3 ± 1.5
Daily hassles	10.7 ± 1.2^*2^	11.8 ± 1.2^**2^	8.4 ± 1.1^**1^	10.7 ± 1.2
Domestic stress	7.7 ± 1.4	8.1 ± 1.4	7.0 ± 1.3	8.8 ± 1.4
Non-domestic stress	11.1 ± 1.9^*1 *2 *3^	7.6 ± 1.9	8.1 ± 1.8	8.8 ± 1.9

multi-comparison by multilevel modeling

^*1 ^p < 0.05 vs. control ^*2 ^p < 0.05 vs. buffer ^*3 ^p < 0.05 vs. pre-migraine

^**1 ^p < 0.01 vs. control ^**2 ^p < 0.01 vs. buffer ^**3 ^p < 0.01 vs. pre-migraine

The stressful event value for migraine days was the highest and showed a significant difference when compared to buffer days. The stressful event value for pre-migraine days was the third highest and showed no significant difference when compared to other days.

The daily hassle value of pre-migraine days was the highest and was significantly higher than buffer days, and it had a higher value than that of migraine days, although the difference was not significant.

There was no significant difference in the values for domestic stress between the day categories. For non-domestic stress, values on migraine days were significantly higher than on other days, and there was no significant difference between pre-migraine days and buffer days or between pre-migraine days and control days.

### 3) Psychological factors (see Table 3)

**Table 3:** Mean scores for psychological factors for each of the days

	Migraine days N = 172 Mean (S.E.) effect	Pre-migraine days N = 128 Mean (S.E.) effect	Buffer days N = 320 Mean (S.E.) effect	Control days N = 265 Mean (S.E.) effect
Headache severity	17.1 ± 1.4^**1 **2 **3^	7.0 ± 1.4^**1^	7.2 ± 1.3^**1^	4.2 ± 1.4
Sleep quality^†^	22.7 ± 2.1	22.8 ± 2.1	22.1 ± 2.0	20.7 ± 2.1
Sleepiness	18.0 ± 1.9	15.5 ± 1.9	16.4 ± 1.8	16.6 ± 1.9
Anxiety	13.8 ± 2.5^*2^	11.8 ± 2.6	11.1 ± 2.5^*1^	14.0 ± 2.5
Depression	10.6 ± 2.3	9.3 ± 2.3	8.5 ± 2.3^*1^	11.0 ± 2.3
Irritability	8.8 ± 1.4	7.9 ± 1.4	6.6 ± 1.3	7.0 ± 1.4

^† ^Data from 43 days of migraine, 32 days of pre-migraine, 80 days of buffer, 66 days of control multi-comparison by multilevel modeling

^*1 ^p < 0.05 vs. control ^*2 ^p < 0.05 vs. buffer ^*3 ^p < 0.05 vs. pre-migraine

^**1 ^p < 0.01 vs. control ^**2 ^p < 0.01 vs. buffer ^**3 ^p < 0.01 vs. pre-migraine

Sleep quality was the worst on control days, followed by buffer days, migraine days, and pre-migraine days. However, there was no significant difference between the day categories. The sleepiness value of migraine days was the highest, although the difference was not significant.

The anxiety value was significantly higher on migraine days compared to buffer days. Also, the anxiety value was significantly higher on control days compared to buffer days.

The tendency for depression was the highest on control days, followed by migraine days, pre-migraine days and buffer days. A significant difference was only observed between buffer days and control days.

Irritability was the most severe on migraine days, followed by pre-migraine days, control days, and buffer days, although there was no significant difference in the values of irritability between the day categories.

## Discussion

### Migraine and stress

Daily hassle values 1–3 days before a migraine attack were the highest and had a significant difference compared to buffer days. This finding supports past reports from the United States and Europe [13-15,21], suggesting that daily hassles increase a few days before migraine attack in Japanese patients as is seen in the United States and Europe.

It was reported that migraine was more closely correlated to daily hassles than stressful events [10]. From this viewpoint, there was a significant difference in the stressful event value between buffer days and control days and between buffer days and migraine days. On the other hand, the daily hassle value 1–3 days before migraine attack was significantly higher compared to that of buffer days, similar to past reports, which suggested that stress increased 1–3 days before migraine attack and that it was more closely related to daily hassles than to stressful events.

In this study, however, the values on migraine days and control days were lower than those on pre-migraines days, but significantly higher than those on buffer days, and there was no significant difference between those on migraine days and control days and on pre-migraine days. Thus, the results of this study showed that daily hassles on pre-migraine days did not affect migraine attack so much as has been reported for the United States and Europe. It will be necessary to do studies with more subjects to verify whether or not the reason for the different results comes from a racial difference between Japanese and Americans and Europeans.

Stress was treated as a whole in the past reports on the relationships between migraine and stress. Stresses were divided into two groups: stressful events and daily hassles. This is the first study that analyzed stress divided into domestic and non-domestic categories. Only non-domestic stress was significantly severe on migraine days compared to other days. In other words, this means that patients with migraines were strongly aware of non-domestic stress on a migraine attack day. However, this result is different from the result that daily hassles were high 1–3 days before migraine attack. Based on the result that domestic stress 1–3 days before migraine attack was not significantly different than on other days, a stress model other than domestic and non-domestic stress may needed.

### Migraine and psychological factors

There are few reports on chronological relationships between migraine and psychological factors, but it has been said that prior to migraine attack, mood changes, such as alertness[11,14,15], tension[13-15], depressive tendency [11-14], irritability[12-15] and fatigue[12-15], occur. However, it has been noted in many recent studies about migraine that migraine is related to psychological factors such as anxiety [22] and sleep conditions [23]. In this study, we assessed not only depressive tendency (as one of the symptoms, not as a major disorder) and irritability, but also anxiety and sleep conditions.

The results of this study showed little difference in sleep quality on migraine days and pre-migraine days. The anxiety value was significantly higher on migraine days compared to buffer days. None of the psychological factor values on pre-migraine days showed a significant difference compared to other days.

It is questionable how stress and mood changes are related to migraine: Are stress and mood changes related to migraine independently or interactively? It was reported that mood changes such as depressive tendency due to stress led to migraine [13]. In the results of this study also, it was found that stress increased 2–3 days before migraine attack, psychological change occurred a little later, and as a result a migraine attack occurred.

### Meaning of results and research prospects

For the prevention of migraine, drug therapy, relaxation, and abstention from alcohol and some kinds of food are recommended. More detailed prevention measures may be possible. This study shows that stress, mainly daily hassles, increase a few days before migraine attack. For example, if a patient feels daily hassles, it may be a sign of a migraine attack. If measures are taken immediately, such as a dose increase or positive escape from stress and stress release through relaxation, the prevention of migraine may be more successful.

### Study limitations

Study limitations include the following: 1) the number of subjects was small; 2) the headache diary period was short, only for two weeks; and 3) the subjects included both working people and housewives, and non-domestic stress was very different between them. To clarify whether or not the results of the study are applicable only to migraine, it is necessary to compare migraine and chronic headache, for which a relation to stress is indicated, such as tension-type headache. It will also be necessary to conduct further studies with larger numbers of subjects to accumulate the needed data.

## Conclusion

1) Psychosocial stress preceding the onset of migraines by several days was suggested to play an important role in the occurrence of migraines. However, stress 2–3 days before migraine attack was not so high as has been reported in the United States and Europe.

2) There were no significant differences in the values of psychological factors between pre-migraine days and other days.

## Competing interests

The authors declare that they have no competing interests.

## Authors' contributions

UY carried out the statistic analysis. AS participated in the design of the study. KH participated in the design of the study. YA participated in the design of the study. MM carried out the revising the manuscript. KY carried out the statistic analysis and the revising the manuscript. KT participated in the design of the study. All authors read and approved the final manuscript.
